# Medical therapy of stricturing Crohn’s disease: what the gut can learn from other organs - a systematic review

**DOI:** 10.1186/1755-1536-7-5

**Published:** 2014-03-29

**Authors:** Dominik Bettenworth, Florian Rieder

**Affiliations:** 1 Department of Medicine B, University Hospital of Münster, Münster, Germany; 2 Department of Gastroenterology and Hepatology, Digestive Disease Institute, Cleveland Clinic Foundation, Cleveland, OH, USA; 3 Department of Pathobiology, Lerner Research Institute, NC22, Cleveland Clinic Foundation, 9500 Euclid Avenue, Cleveland, OH 44195, USA

**Keywords:** Crohn’s disease, Intestinal fibrosis, Organ fibrosis, Anti-fibrotic agents

## Abstract

Crohn’s disease (CD) is a chronic remitting and relapsing disease. Fibrostenosing complications such as intestinal strictures, stenosis and ultimately obstruction are some of its most common long-term complications. Despite recent advances in the pathophysiological understanding of CD and a significant improvement of anti-inflammatory therapeutics, medical therapy for stricturing CD is still inadequate. No specific anti-fibrotic therapy exists and the incidence rate of strictures has essentially remained unchanged. Therefore, the current therapy of established fibrotic strictures comprises mainly endoscopic dilation as well as surgical approaches. However, these treatment options are associated with major complications as well as high recurrence rates. Thus, a specific anti-fibrotic therapy for CD is urgently needed. Importantly, there is now a growing body of evidence for prevention as well as effective medical treatment of fibrotic diseases of other organs such as the skin, lung, kidney and liver. In face of the similarity of molecular mechanisms of fibrogenesis across these organs, translation of therapeutic approaches from other fibrotic diseases to the intestine appears to be a promising treatment strategy. In particular transforming growth factor beta (TGF-β) neutralization, selective tyrosine kinase inhibitors, blockade of components of the renin-angiotensin system, IL-13 inhibitors and mammalian target of rapamycin (mTOR) inhibitors have emerged as potential drug candidates for anti-fibrotic therapy and may retard progression or even reverse established intestinal fibrosis. However, major challenges have to be overcome in the translation of novel anti-fibrotics into intestinal fibrosis therapy, such as the development of appropriate biomarkers that predict the development and accurately monitor therapeutic responses. Future clinical studies are a prerequisite to evaluate the optimal timing for anti-fibrotic treatment approaches, to elucidate the best routes of application, and to evaluate the potential of drug candidates to reach the ultimate goal: the prevention or reversal of established fibrosis and strictures in CD patients.

## Methods

### Literature search and data selection

A comprehensive literature search was performed to assess all relevant citations found in Embase, Medline (service of the US National Library of Medicine (NLM) and the National Institutes of Health (NIH)) and the Cochrane Library for the following key words: (‘Crohn’s disease (CD’) OR ‘Crohn’s’ AND (‘stricture’ OR ‘fibrosis’), (‘kidney’ OR ‘liver’ OR ‘skin’ OR ‘lung’ OR ‘systemic nephrogenic’ AND ‘fibrosis’ OR ‘anti-fibrotic therapy’). Additionally, references of cited original articles and reviews were further assessed for relevant work. The search included studies between 1960 and 2013. These data together with the authors’ personal experience in the field represent the basis of this review.

## Introduction

**Figure 1:**
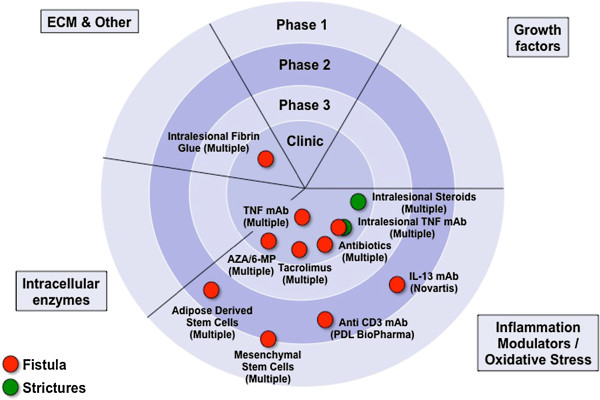
**Therapeutic strategies to modify wound healing in Crohn’s disease.** Currently available therapies for stricturing or fistulizing CD are depicted. Data derived from Embase, Medline and ClinicalTrials.gov. 6-MP, 6-mercaptopurine; AZA, azathioprine; CD, Crohn’s disease; ECM, extracellular matrix; IL, interleukin; mAb, monoclonal antibody; TNF, tumor necrosis factor.

Crohn’s disease (CD) is a chronic remitting and relapsing disease [1]. During acute flares, CD patients may present with mainly inflammation driven symptoms such as diarrhea, abdominal pain and weight loss [2]. However, over the long-term, the naturally progressive disease course often culminates in stricture formation. For example, around 40% of CD patients with ileal disease develop clinically apparent strictures [3]. Strictures may be subdivided into fibrotic and inflammatory as well as mixed forms [4]. Accordingly, strictures including inflammatory alterations might benefit from anti-inflammatory therapy through a reduction of the inflammation-mediated edema [5]. During the last two decades, the therapeutic armamentarium for CD has expanded significantly, especially with the use of anti-tumor necrosis factor alpha (TNF-α)-based strategies that can lead to sustained clinical response rates in a substantial proportion of CD patients [6-8]. The success of anti-TNF antibodies fueled the hope for altering the natural course of CD. Most recent epidemiological data, however, revealed that despite the establishment of early immunosuppressive therapy in CD patients with an increased risk of disabling disease, the frequency of fibrostenosing complications did not significantly change [9]. Thus, a specific anti-fibrotic therapy for stricturing complications in CD patients is needed. Despite recent advances in the pathophysiological understanding of intestinal fibrosis in CD [10,11] and in contrast to fibrotic complications in other organs, no specific anti-fibrotic drugs for intestinal strictures are currently available, and all existing therapies used in clinically apparent CD-associated stenosis are the same that are prescribed for active luminal disease. The same holds true for the treatment of penetrating CD, another inflammatory bowel disease (IBD)-associated complication that is linked to impaired intestinal remodeling and healing. Available drugs for the treatment of fibrostenosing or penetrating IBD are depicted in Figure 1. Consequently, the therapy of choice for fibrostenosing CD, in conjunction with purely anti-inflammatory therapy, comprises endoscopic dilation (ED) procedures as well as surgical approaches, with all their associated limitations and morbidity [12-14]. A significant number of patients have to undergo multiple surgeries, with the subsequent risk of developing intestinal failure. In general, isolated strictures with a length of 4 cm or less [12] which are devoid of ulcers [15] and are accessible by colonoscopy [16] or double-balloon enteroscopy [17] qualify for ED. Although ED procedures for stricturing CD are usually technically successful, more than one third of patients will still undergo surgery within the next years due to insufficient response to ED [12-14]. In addition, major complications such as bowel perforation, bleeding or infection are reported in a range of 2 to 5% [12,18]. In those CD patients where endoscopic stricture therapy is technically not feasible or not indicated, surgical approaches including resection and strictureplasty are recommended. While repeated surgical bowel resections bear the risk for induction of deficiencies in gastrointestinal functions and ultimately may manifest short bowel syndrome and intestinal failure, strictureplasty can treat intestinal obstruction without reducing intestinal length [10]. Here, the incidence of major complications including anastomotic leakage, abscess, fistula or sepsis is present in about 6% [19]. The recurrence rates differ between various forms of strictureplasty from 23 to 41% [20-22]. To date, no head to head comparison between ED and strictureplasty has been performed yet. Taken together, the insufficient therapeutic impact of currently available anti-inflammatory drugs on stricture prevention and treatment, the complications associated with ED or surgical treatment approaches associated with high socioeconomic burden [23] as well as the high recurrence rates of stricturing CD after procedures demand the development and evaluation of specific anti-fibrotic agents for stricturing CD.

## Review

### A basic overview of mechanisms of intestinal fibrosis

Fibrosis is defined as the accumulation of collagen-rich extracellular matrix (ECM) in response to tissue damage and is a common complication of multiple chronic diseases [24]. Repetitive or persistent injury of the intestinal epithelium and subsequently deeper layers of the intestinal wall may initiate, perpetuate or maintain progressive fibrosis [24]. With regard to the gastrointestinal tract, acute, short-lived epithelial damage may occur as a consequence of peptic ulcers, infectious enteritis or mild diverticulitis, leading to a full restitution of tissue structure. In contrast, in CD, the gastrointestinal mucosa is exposed to chronic remitting or continuous pro-inflammatory and environmental stimuli. Pleiotropic mechanisms are activated like cellular stress, increased production of inflammatory cytokines and chemokines such as IL-13 or IL-17 [25,26] and growth factors, such as transforming growth factor beta 1 (TGF-β1) [27], insulin-like growth factor (IGF) [28], platelet-derived growth factor (PDGF) [29] and basic fibroblast growth factor (bFGF) [30]. These mediators crucially contribute to morphological and functional alterations within the bowel wall that may finally culminate in stricture formation and loss of physiological gut functions [31,32]. In the murine model of 2,4,6-trinitrobenzene sulfonic acid (TNBS)-induced colitis, inhibition of IL-13 signaling by administration of small interfering RNA targeting the IL-13-α2 receptor attenuated inflammation-associated intestinal fibrosis [25]. This observation was corroborated by work from the same group, indicating that TGF-β1 secretion by macrophages was increased upon IL-13 stimulation *in vitro* and *in vivo*[33]. Contractility of isolated intestinal smooth muscle from CD patients was enhanced after pre-stimulation with IL-13 [34]. Additionally, increased IL-13 transcripts were detected in muscle extracts from intestinal samples of fibrotic CD patients compared to samples from non-inflamed areas, which results in inhibition of fibroblast matrix metalloproteinase (MMP) synthesis [35]. In contrary to these findings, there was no difference in IL-13 production in mucosal explants and lamina propria mononuclear cells between patients with stricturing CD and control subjects in a different study [36].

IL-17A was found to possess pro-fibrotic activity in various cell types including cardiac fibroblasts [37], hepatic stellate cells [38], skin fibroblasts [39] and lung epithelial cells [40]. In addition, IL-17E was shown to increase collagen production in lung fibroblasts [41]. Consistent with a role of IL-17 in fibrosis IL-17 tissue levels were increased in a murine model of intestinal fibrosis [26]. In human fibrotic CD, IL-17A, but not IL-17E, was overexpressed within tissue samples from CD strictures as compared to non-strictured CD areas and healthy gut. IL-17 secretion from cultured intestinal explants from strictured CD patients was significantly increased as compared to non-strictured CD samples. Moreover, myofibroblasts from CD strictures, expressing the IL-17A receptor, generated more collagen and tissue inhibitor of metalloproteinase 1 (TIMP-1) and revealed inhibitory effects on myofibroblast migration [42]. In a clinical trial of patients with inflammatory CD, however, blockade of anti-IL-17A by administration of the anti-IL-17A antibody secukinumab failed to improve disease activity, was associated with a high rate of serious adverse events and had to be stopped prematurely since predefined criteria for futility were met [43], indicating that further studies are necessary before using anti-IL-17-based strategies in the therapy of intestinal fibrosis.

The core mediator in various organs for both, the initiation as well as the maintenance of fibrosis is TGF-β [44]. This growth factor is produced by a vast majority of cells and organs in mammals and is stored in large amounts extracellularly through chemical cross-links to the ECM [45]. The TGF-β/Smad signaling pathway appears pivotal for the development of fibrosis [44]. Canonical intracellular signal transduction is mediated by Smad2/3 phosphorylation by TGF-β receptor I kinase leading to binding of Smad4. This complex translocates into the nucleus and induces TGF-β-specific pro-fibrotic gene expression [46]. Inhibitory members of the Smad family such as Smad6/7 block the phosphorylation of Smad2/3 via competition with the TGF-β receptor I kinase [47,48].

In addition to the above mentioned mediators, the imbalance of MMPs and TIMPs, which are physiologically involved in maintaining a state of ‘healthy’ remodeling and restitution, can aggravate structural changes of the bowel wall [48,49].

Restoring the integrity of the intestinal barrier, culminating in epithelial wound closure may help to resolute and regress fibrosis, since continuous barrier defects appear to be one potential trigger for chronic inflammation promoting pro-fibrotic alterations. To this aim ECM-producing mesenchymal cells are recruited. These cells may migrate from neighboring tissue [50], originate from circulating mesenchymal cell precursors or bone marrow stem cell-derived mesenchymal cells [51], arise by proliferation from existing mesenchymal cells [30] or result from epithelial- or endothelial-mesenchymal transition (EMT and EndoMT, respectively) [52]. Recently, the intestinal microbiota has been identified as a key pro-fibrotic factor, as suggested by several lines of evidence: 1) Ligands to Toll-like receptor 4 (TLR4) (predominantly from gram-negative bacteria) or TLR2 (predominantly from gram-positive bacteria) activate NF-κB, resulting in cytokine and chemokine secretion by intestinal mesenchymal cells [53]. 2) In several experimental colitis models, microbes initiate or perpetuate gut inflammation and fibrosis, such as in SAMP1/YitFc mice, the IL-10 knock out mice, TNBS and peptidoglycan-polysaccharide (PG-PS)-induced colitis [54]. 3) In humans, gene variants that affect innate immunity, located in or near genes involved in bacterial recognition and processing, are genetically associated to IBD or CD as well as complicated CD courses [55]. 4) Finally, circulating antibodies against microbial components are commonly found in IBD patients and are believed to arise from an immune response towards the luminal microbiota. These antibodies are qualitatively and quantitatively associated with and predictive of a more complicated disease phenotype including fibrostenosis [50,56-58].

Beside chronic inflammation as a major driver of intestinal fibrosis, inflammation-independent mechanisms deserve closer attention. In particular, activated mesenchymal cells, also referred to as disease-activated myofibroblasts, produce and secrete high levels of several collagen types, such as type I, III and V [59-61] and ECM compounds, such as fibronectin or tenascin C [62,63] which get deposited, linked and subsequently form matrix networks that can lead to increased tissue stiffness [64]. Stiffness in itself in the absence of inflammation activates further mesenchymal cells in the form of a positive feedback loop [64]. The ability of myofibroblasts to contract might further increase the luminal narrowing of the intestine and contraction can be induced by factors other than inflammatory mediators [65]. Interestingly, latent matrix-bound TGF-β1 can be activated by mesenchymal cells’ traction forces, pulling against a mechanically resistant ECM. This leads to a conformation change of the latency-associated peptide liberating the active TGF-β1 [66].

In summary, despite different physiological functions and unique features of the human gut, such as the high load of microbial components, intestinal fibrosis shares pathological core features with fibrosis of other organs, such as the lung, kidney, skin or liver [24]. Consequently, anti-fibrotic agents with proven efficacy in fibrotic disease of these organs may represent promising candidates for stricturing CD and will be discussed in the following section.

### Anti-fibrotic therapeutic approaches in other organs

Commonly used drugs for the anti-inflammatory therapy of CD have been observed to possess at least minor anti-fibrotic properties in other organs. For example, corticosteroids were found to decrease pro-collagen expression *in vivo* and *in vitro*[67] as well as to inhibit collagenase activity [68]. Corticosteroids show some effect in retroperitoneal fibrosis [69], systemic sclerosis [70] and idiopathic pulmonary fibrosis (IPF) [71]. In contrast, human intestinal myofibroblasts respond to corticosteroids with enhanced pro-collagen expression upon dexamethasone stimulation [72]. In stricturing CD, small case series report variable therapeutic success rates of intralesional steroid injection [73]. The long-term systemic administration of corticosteroids in CD, however, is obsolete due to severe and pleiotropic side effects. Azathioprine, one of most commonly prescribed immunosuppressive drugs for maintenance of remission in CD patients, is beneficial in the treatment of retroperitoneal fibrosis [74] and fibrotic pulmonary disease [75,76]. In CD patients, azathioprine may delay postoperative, fibrotic complications [77], however, the early use of immunosuppressive treatment regimens does not reduce the occurrence of intestinal strictures and frequency of surgical interventions in the long-term [9,78]. TNF-α is a critical cytokine in the pathogenesis of IBD and to date four anti-TNF antibodies have shown clinical efficacy as anti-inflammatory agents [79-82] and are available for clinical use. Several reports from liver fibrosis [83], pulmonary fibrosis [84] and systemic sclerosis [85] suggest an anti-fibrotic effect of anti-TNF treatment. This could be explained by TNF-α-mediated myofibroblast activation, increased collagen production and TIMP-1 expression as well as inhibition of MMP-2 activity and collagen degradation [86]. In contrast, human intestinal myofibroblasts from CD patients show increased expression of TIMP-1 and decreased collagen production upon exposure to infliximab [87]. After initially conflicting data in patients with CD with a possible pro-fibrotic effect of anti-TNF therapy *in vivo*, recent data revealed no link between anti-TNF administration with intestinal stricture formation [88]. Information derived from a small retrospective study points towards at least a partial amelioration of stricture formation with anti-TNF therapy [89] and anti-TNF treatment may delay the time to surgery in CD [90].

In daily clinical practice, most CD patients present with established strictures, representing the end-stage of the fibrotic process, and most clinicians see this scenario as an inevitable progression to a likely surgical intervention. This does not have to be so, as the theoretical and practical feasibility to stop or even reverse intestinal fibrosis is supported by the observation that tissue alterations in experimental models of intestinal fibrosis disappear after elimination of the pro-fibrotic stimulus [54]. No therapeutic medical strategy for pre-existing intestinal strictures exists at the moment, but this clinical scenario could greatly improve by looking at existing knowledge derived from other organ systems.

**Figure 2:**
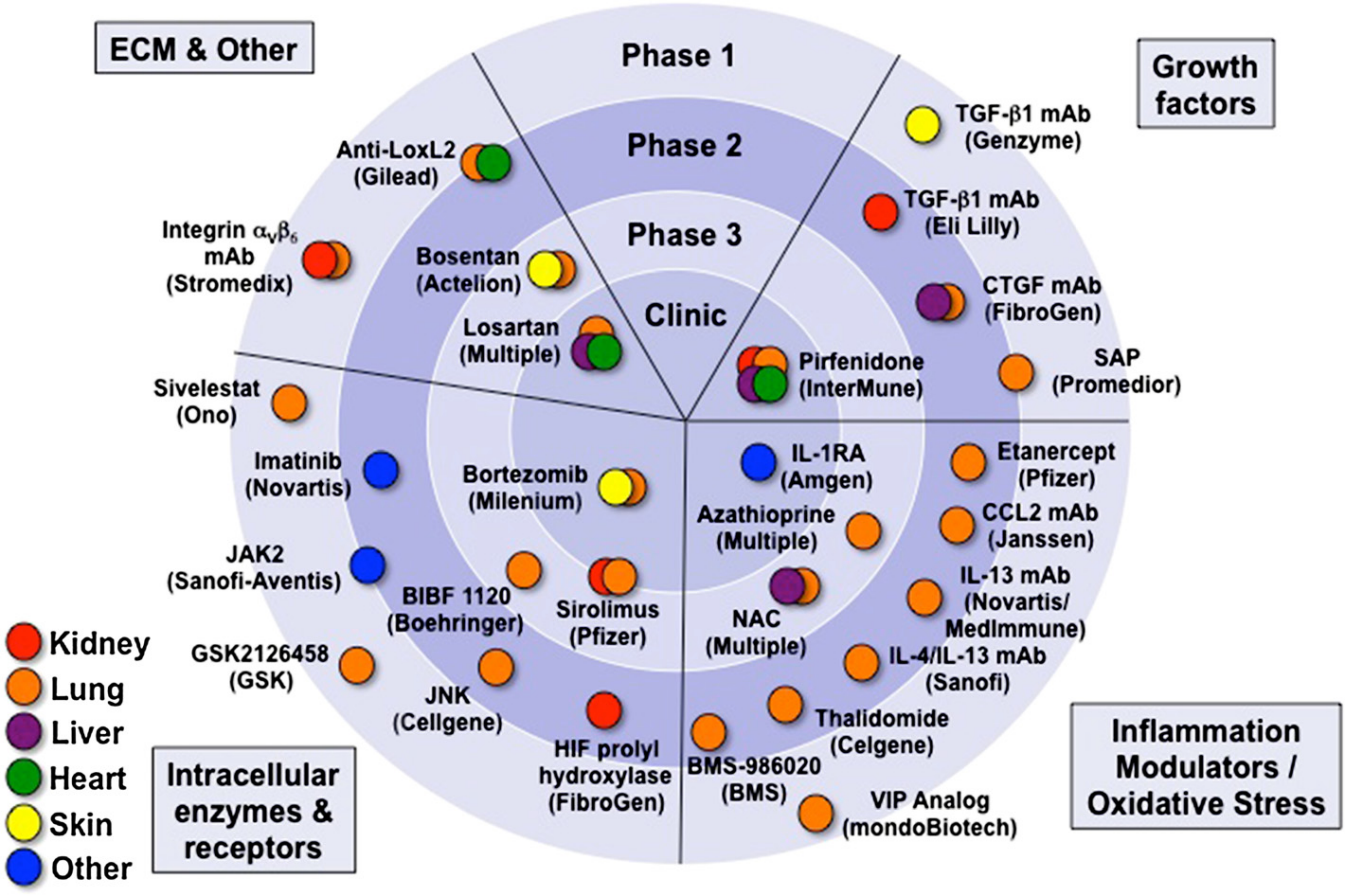
**Pipeline for therapeutic strategies to alter and improve fibrosis in organs other than the intestine.** Each color represents a particular organ. Agents are classified based on their mode of action. Data derived from Embase, Medline and ClinicalTrials.gov. CCL2, chemokine ligand 2; CTGF, connective tissue growth factor; ECM, extracellular matrix; HIF, hypoxia inducible factor; IL, interleukin; JAK, janus kinase; JNK, c-Jun N-terminal kinase; mAb, monoclonal antibody; SAP, serum amyloid P component; TGF-β, transforming growth factor beta; VIP, vasoactive intestinal peptide.

Anti-fibrotic strategies used in the kidney, lung, liver, heart or skin can be grouped into strategies modulating growth factors, inflammation or oxidative stress, intracellular enzymes, and ECM production or assembly (Figure 2). We herein provide a summary of currently available experimental anti-fibrotics that have been tested for indications outside of the intestine. We selected promising mechanisms and agents that could be applicable to stricture therapy of CD in the near future and describe them in greater detail.

### Growth factors

Feasibility of TGF-β1 blockade to treat fibrotic diseases has been shown in IPF. In this condition, TGF-β is driving fibrotic alterations and epithelium-dependent fibroblast activation [44]. In murine models of IPF, antagonizing TGF-β action via Tβ-RII (as a ligand decoy), P144, an inhibitor of TGF-β activity, or P17, an anti-TGF-β peptide, resulted in potent anti-fibrotic effects [91-93]. In human IPF patients, two phase III trials have shown that treatment with pirfenidone, an agent inhibiting TGF-β activity and p38 mitogen-activated protein kinase (MAPK) signaling, improved pulmonary function [94,95]. Pirfenidone is expected to be approved for IPF in Europe soon [96]. Another example for the potential of anti-TGF-β-based strategies is scleroderma, a disease characterized by enhanced expression of TβRI and TβRII in fibroblasts leading to increased collagen I production and ECM deposition in the skin and internal organs [97]. Topical treatment with P144 has shown efficacy in patients with systemic sclerosis, and multiple trials with this agent are currently ongoing. Fresolimumab (GC1008) is a humanized antibody targeting anti-TGF-β1,2 and 3 and was recently evaluated in a phase I trial in patients with treatment-resistant primary focal segmental glomerulosclerosis [98]. The humanized αVβ6 integrin-blocking antibody STX-100, which inhibits the activation of latent TGF-β on epithelial cells, is currently being examined in a phase II trial in IPF patients [24,99]. Comparable results have been reported from kidney fibrosis: preclinical *in vitro* and *in vivo* studies indicate an anti-fibrotic activity using anti-TGF-β antibodies, soluble TGF-β receptor, blockade of TGF-β activation by decorin, a small-molecule inhibitor of TGF-β receptors [100], administration of inhibitory protein Smad7 [101] and thrombospondin-1 (THBS-1) blocking agent inhibiting TGF-β activation [102]. In human patients with focal segmental glomerulosclerosis, a phase I trial with fresolimumab revealed promising results [98]. Moreover, hepatic growth factor, acting as an inhibitor of Smad2/3 translocation in fibroblasts [103] was observed to mediate anti-fibrotic effects in experimental models of renal and liver fibrosis but was also associated with an increased risk of hepatocellular carcinoma [104]. Substrate specificity of therapeutics needs to be taken into consideration as well. In an experimental model of scleroderma, blocking activin receptor-like kinase 5 (ALK5), which is involved in phosphorylation of Smad2/3, leads to decreased fibroblast activation. However, ALK5 blockade in clinical trials was associated with adverse events due to cross-reactivity with other kinase inhibitors [105].

In addition to the Smad-signaling cascade, non-Smad pathways comprising TGF-β1 activated MAPKs and several tyrosine kinases have been targeted for anti-fibrotic actions. For example, c-Abelson (c-Abl), a component of the Bcr-Abl oncogene, can be effectively blocked by selective tyrosine kinase inhibitors such as imatinib. This agent inhibits PDGF as well and thus potentially regulates fibroblast proliferation and transformation [106]. Despite promising results from *in vitro* and *in vivo* studies, to date limited experience with tyrosine kinases in human fibrotic diseases is available [107,108]. Of note, novel tyrosine kinase inhibitors such as nilotinib and dasatinib mediate dose-dependent decreases in ECM production and reveal even greater efficacy as compared to that of imatinib [109], while being well tolerated by the patients [110]. In contrast to TGF-β1, other members of the TGF family such as TGF-β3 possess anti-fibrotic properties. Avotermin is a recombinant bioactive human TGF-β3 that has been tested for treatment of dermal scars and significantly reduces the scar size by intradermal injection therapy [111]. In addition, further growth factors such as serum amyloid P (SAP) have been proven effective in experimental models [112,113] of fibrosis and have already entered phase I clinical trials in human patients [114].

The scientific rationale to target TGF-β signaling in stricturing CD comes from *in vitro* as well as *in vivo* observations. For example, intestinal TGF-β overexpression in mice leads to colonic fibrosis and obstruction [27], while disruption of the TGF-β/Smad signaling cascade protects animals from intestinal fibrosis [115]. In human tissue samples from colonic CD strictures, TGF-β and its receptors as well as pSmad2/3 expression are increased, while Smad7 expression was significantly reduced [116]. Although targeting TGF-β signaling for fibrotic diseases has a strong scientific rationale, it has to be taken into account that this growth factor is not only crucially involved in fibrogenesis but additionally functions as a key regulator of cellular processes including differentiation, proliferation, transformation, tumor suppression as well as immunoregulation and its actions may be context-dependent [96,117]. For example, TGF-β1-deficient mice develop severe multiorgan inflammation and expire by 5 weeks of age [118,119]. This outcome occurs even under germ-free conditions [120] and is mediated by CD4^+^ T cells [121]. Similarly, targeted deletion of Smad2 and Smad4 is associated with early death in mice [122,123]. Furthermore, administration of metelimumab, a monoclonal antibody against TFG-β1, in human systemic sclerosis patients was associated with significantly more serious adverse events than placebo treatment including musculoskeletal pain, progression of skin involvement and death [124]. Possible side effects during anti-TGF-β therapy would have to be carefully monitored, in particular in case of pre-existing inflammation. Therefore, neutralizing TGF-β 1 *in vivo,* as an anti-fibrotic approach in CD may be highly problematic, as this may actually lead to disease exacerbation given the potent anti-inflammatory and immunoregulatory properties of this cytokine.

### HMG-CoA reductase inhibitors

HMG-CoA reductase inhibitors were developed with the intention to decrease cholesterol levels. More recently, they were found to mediate anti-inflammatory as well as anti-fibrotic effects *in vitro*[125], including decreased proliferation of mesangial cells, lower fibronectin as well as type IV collagen expression and decreased secretion of TGF-β1 and connective tissue growth factor (CTGF) [126-128]. Corroborating these *in vitro* findings, HMG-CoA reductase inhibitors revealed various anti-fibrotic effects in murine models of nephropathy and fibrosis [126,127]. In CD patients, HMG-CoA reductase inhibitor atorvastatin was shown to mediate anti-inflammatory effects such as inhibition of T cell recruitment via reduced CXCL10 levels [129] and reduce surrogate inflammatory markers such as calprotectin, C-reactive protein and TNF-α expression [130]. Furthermore, it was demonstrated that simvastatin reduced TGF-β1 expression in human fibroblasts by inhibition of Smad3 phosphorylation [131] leading, together with induced apoptosis in fibroblast and myofibroblasts, to a significant amelioration of experimental fibrosis [132]. In addition, mesenchymal cells isolated from patients with radiation-induced intestinal fibrosis respond to pravastatin treatment with significantly decreased production of fibronectin and type-1 collagen through Rho-/ROCK-dependent reduction of CTGF expression [133]. Nevertheless, the exact anti-fibrotic potential of statin treatment in stricturing CD still needs to be defined by the use of hard clinical endpoints, but this drug class has an already established safety profile for routine clinical use and could serve as a potential anti-fibrotic treatment approach.

### Renin-angiotensin system (RAS) modulators

Angiotensin II (AT II) is the major mediator of the renin-angiotensin system (RAS). AT II may increase ECM accumulation through plasminogen activator inhibitor-1-mediated decrease of MMPs and enhance TGF-β1 production in cardiac and renal fibrosis [134,135]. The impact of AT II can also be observed in liver fibrosis. In hepatic stellate cells, AT II induces contraction and proliferation accompanied by increased collagen and TGF-β expression [136]. Accordingly, progression of liver fibrosis in hepatitis C virus positive patients is significantly decreased after treatment with angiotensin-converting enzyme inhibitors [137]. AT II is crucially involved in the manifestations of renal fibrosis by induction of pro-fibrotic effector molecules and EMT resulting in enhanced ECM production [138], and inhibition of AT II using angiotensin-converting enzyme (ACE) inhibitors or blocking agents towards the AT I receptor has emerged as a therapeutic approach to slow down renal disease progression [139] and revealed anti-fibrotic actions in the lung, heart and liver [140-143]. Interestingly, all components of the RAS have been detected in the human colonic mucosa [144] and AT II is increased in the mucosa of CD patients [145]. *In vivo* administration of the ACE inhibitor enalaprilate has been proven to reduce weight loss and histological damage in murine dextran sulfate sodium (DSS)-induced colitis [146]. ACE inhibitor treatment was also effective in spontaneous colitis of IL-10-deficient mice [147] and this finding has been confirmed by other studies [148,149].

Of note, through its AT1 receptor, AT II enhances the expression of CTGF, and administration of AT II inhibitors and AT1 receptor antagonists significantly ameliorates or reverses fibrotic alterations in experimental colitis reflected by reduced collagen amounts and TGF-β1 mRNA levels [150,151]. Existing preclinical data in IBD combined with clinical trials from the liver and kidney make RAS modulation a promising future approach for CD-associated fibrosis.

### Inflammation modulators

Pro-inflammatory cytokines contributing to the pathogenesis of IBD could also be involved in the development of intestinal fibrosis in CD. For example, IL-1 modulates myofibroblast activation, chemokine production, MMP secretion [152] and is involved in EMT induction [153]. IL-6 is known to regulate TGF-β and TGF-βR2 expression as well as fibroblast proliferation [154,155] and is strongly upregulated in serum and tissue samples from CD patients [156]. IL-4 and IL-13 represent pivotal mediators of immune activation and T helper cell 2 responses are crucially involved in the development of intestinal fibrosis *in vivo*. IL-13 mediates, through binding to its IL-13Rα, an increased production of TGF-β and is a key player in the initiation of fibrotic alterations in the intestine [25]. Confirmatively, antagonism of IL-13 is effective to prevent fibrosis development in experimental colitis [33,157]. In addition, several IL-13 antibodies such as lebrikizumab, tralokinumab and QAX576 as well as the anti-TNF antibody etanercept and the immunomodulatory drug thalidomide are currently being evaluated for their anti-fibrotic potency in liver fibrosis and pulmonary fibrosis [24,158-160].

The process of inflammation and fibrosis are likely to be intertwined through angiogenesis and lymphangiogenesis. Increased levels of factors implicated in angiogenesis have been documented in IBD patients, such as vascular endothelial growth factor A (VEGF-A) [161]. At a cellular level, PDGF increases proliferation and migration of fibroblasts and myofibroblasts [46]. In the human intestine, PDGF facilitates ECM deposition and is upregulated in inflamed colonic tissue specimen of CD patients [29]. Experience with blocking these agents in other fibrotic diseases exist. In a phase II trial, combined blockade of VEGF, PDGF and bFGF by the indolinone derivative BIBF 1120 tends to decrease the development of human IPF [162]. Critical for the future use of inflammation modulators in the therapy of CD-associated fibrosis will be the quality, quantity and the timing of the approach because all of the above mediators act at different times throughout the disease course, in differing combinations and quantities [163]. Additionally, all of the above molecules interact with each other and blocking a single cytokine at a specific time might not be sufficient for effective anti-fibrotic therapy.

### Extracellular matrix modulators

The imbalance between deposition and degradation of ECM in fibrotic disease is a logical target for anti-fibrotic treatment approaches. Stimulation of MMPs as central regulators of ECM disassembly were expected to reverse fibrotic alterations, however, clinical studies in patients with nephrosclerosis failed to show efficacy [164]. Likewise, depletion of TIMP should decrease fibrotic changes, but no amelioration of renal fibrosis was observed following TIMP inhibition in mice [165]. With regard to the intestine, there is growing evidence for MMPs as a regulator of intestinal barrier function and mucosal defense [166], indicating pleiotropic functions of this molecular group in addition to purely matrix regulation. For example, serum MMP-9 levels correlate with disease activity in pediatric CD patients [167] and may be used as a biomarker to follow the course of disease in adult CD patients as well [168,169]. Colonic tissue expression of MMP-1, MMP-2, MMP-3 and MMP-9 was significantly increased in samples from inflamed mucosa as compared to non-inflamed mucosal samples [170]. Increased MMP-9 expression in inflamed tissue colonic specimen from CD patients seems to be associated with decreased likelihood of disease recurrence [171]. Finally, in mucosa specimen overlaying colonic strictures in CD patients, MMP-3 and MMP-12 expression was significantly reduced [116]. Thus, MMPs need to be further and carefully investigated as possible targets for anti-fibrotic treatment in as well as outside of the intestine and are not yet ready for prime time.

### Intracellular enzymes and receptors

The mammalian target of rapamycin (mTOR) protein is a serine/threonine protein kinase that consists of several complexes among which mTOR complex 1 regulates protein synthesis, proliferation as well as fibrotic actions [46]. mTOR inhibitors possess direct anti-fibrotic properties by decreasing fibroblast and myofibroblast numbers and by reducing pro-fibrotic cytokine expression, including IL-4, IL-6, IL-13, IL-17, TGF-β1 as well as type I and III collagen [172,173]. Efficacy of mTOR inhibitors have been demonstrated in numerous fibrotic disorders of the skin, lung, kidney and liver [46]. With regard to CD, a randomized, double-blind clinical trial found that everolimus was as effective as azathioprine to achieve steroid-free remission in 138 patients with active CD [174]. Additionally, there are two case reports indicating that mTOR inhibitors sirolimus and everolimus are able to induce remission in refractory CD [175,176]. Given the fact that mTOR inhibitors possess anti-fibrotic as well as immunosuppressive effects, this class of drug appears to be promising for intestinal fibrosis therapy, however, the definitive therapeutic potential for intestinal fibrosis remains to be defined yet.

Peroxisome proliferator-activated receptor gamma (PPAR-γ) is a nuclear receptor that modulates gene expression and is involved in various physiological and pathological processes including inflammation and fibrosis [177]. After stimulation with specific ligands, PPAR-γ directly antagonizes Smad3 or reduces CTGF expression [178]. PPAR-γ agonists are able to improve experimental fibrosis, while PPAR-γ selective antagonists abolish anti-fibrotic actions [179,180]. Given the fact that CTGF is a key downstream effector of TGF-β on connective tissue cells, FG-3019, a humanized antibody targeting CTGF, has been developed and successfully passed phase I trials in several fibrotic disorders and recently entered phase II studies [24]. In the human intestine, PPAR-γ has been detected in the colonic mucosa and has been identified as a mediator of established anti-inflammatory drugs such as 5-ASA [177,181]. The future role of PPAR-γ agonists as a possible target for anti-fibrotic treatment in stricturing CD is promising, given the combined action against inflammation and fibrosis and its well-defined mechanism of action. Furthermore, other drug candidates such as endothelin A receptor antagonist bosentan have shown promising results in patients with IPF and renal interstitial fibrosis and deserves further investigation [182,183].

**Figure 3:**
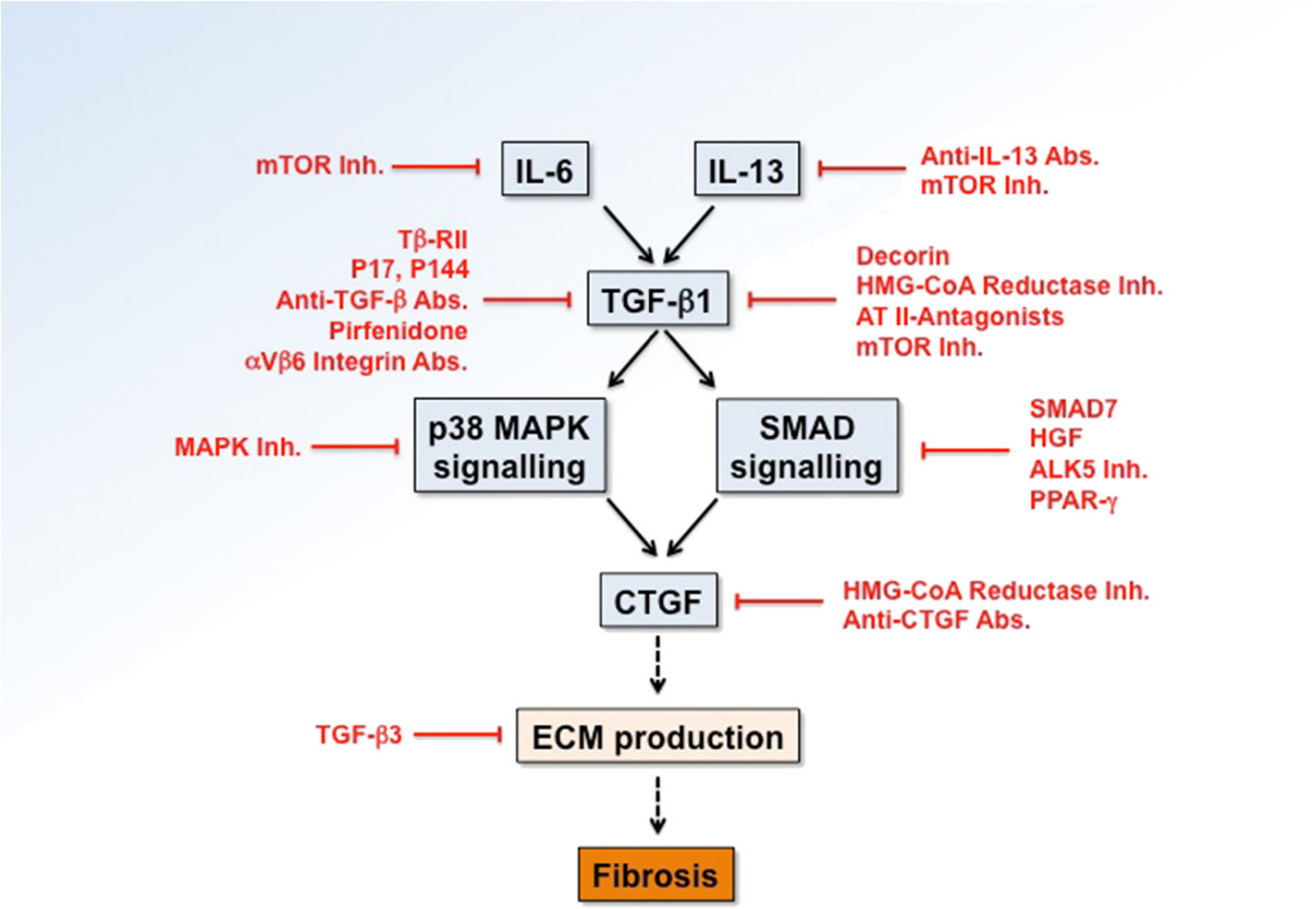
**Compounds used as anti-fibrotic therapies in other organs and their mode of action.** The blue boxes represent a major profibrotic pathway. Compounds are depicted in red, indicating their mechanism of action. Abs, antibodies; ALK, activin receptor-like kinase; AT, angiotensin; CTGF, connective tissue growth factor; ECM, extracellular matrix; HGF, hepatocyte growth factor; HMG-CoA, 3-hydroxy-3-methylglutaryl-coenzyme A; Inh, Inhibitor; IL, interleukin; MAPK, mitogen-activated protein kinase; mTOR, mammalian target of rapamycin; PPAR, peroxisome proliferator-activated receptor; SMAD, small mothers against decapentaplegic; TGF, transforming growth factor.

A summary of compounds used as anti-fibrotic therapies in other organs and their mode of action is depicted in Figure 3. Anti-fibrotic clinical trials of major interest are shown in Table 1.

**Table 1:** Completed clinical trials evaluating anti-fibrotic drugs in organs other than the gut

**Parameter**	**Author**	**Study organ**	**Number of patents**	**Type of trial**	**Study drug**	**Mode of action**	**Outcome measures**
Growth factors	Noble [95]	Lung	779	RCT, phase III	Pirfenidone	Inhibition of TGF-β	Increased FVC in IPF
	Trachtman [98]	Kidney	16	Open-label, phase I	Fresolimumab	Antibody targeting all isoforms of TGF-β	Safety, pharmacokinetics
	Ferguson [111]	Skin	223	RCT, phase I/II	Avotermin	Antibody targeting TGF-β3	Acceleration and permanent improvement in dermal scaring
	Dillingh [114]	Lung	29	RCT, phase I	rhSAP	Substitution of SAP	Reduction in SAP levels and circulating fibrocytes in healthy control and IPF patients
Oxidative stress	Raghu [158]	Lung	88	RCT, phase II	Etanercept	Blockade of TNF	Physiological and functional decrease in disease progression in IPF
	Corren [159]	Lung	219	RCT, phase II	Lebrikizumab	Antibody targeting IL-13	Improved lung function in asthmatic patients
	Horton [160]	Lung	23	RCT, phase III	Thalidomide	Anti-angiogenic and anti-inflammatory	Improvement of cough and respiratory quality of life in IPF
Intracellular enzymes and receptors	Richeldi [162]	Lung	432	RCT, phase II	BIBF 1120	Tyrosine kinase inhibitor	Tendency towards reduced decline of lung function in IPF
	Daniels [108]	Lung	119	RCT, phase II/III	Imatinib	Tyrosine kinase inhibitor	No effect on survival and lung function
ECM and other	Couluris [140]	Lung	20	Uncontrolled, interventional study, phase II	Losartan	AT1 antagonist	Stabilization of lung function in IPF
	el-Agroudy [142]	Kidney	162	RCT, phase II	Losartan	AT1 antagonist	Decreased TGF-β1 plasma levels and proteinuria in renal interstitial fibrosis
	Kuhn [182]	Skin	10	Prospective, open-label, phase II	Bosentan	Endothelin receptor antagonist	Reduced skin thickening in systemic sclerosis
	Diez [141]	Heart	34	Uncontrolled, phase II	Losartan	AT1 antagonist	Decreased myocardial collagen content and left ventricular chamber stiffness in hypertensive patients
	De [143]	Liver	39	RCT, phase II	Losartan	AT1 antagonist	Reduction of portal pressure in patients with liver cirrhosis
	King [183]	Lung	616	RCT	Bosentan	Endothelin receptor antagonist	Improvement of FVC and diffusing capacity in IPF

AT1, angiotensin II receptor antagonist subtype 1; CXCL, CXC ligand; ECM, extracellular matrix; FVC, forced vital capacity; IL, interleukin; IPF, idiopathic pulmonary fibrosis; RCT, randomized controlled trial; SAP, serum amyloid P component; TGF-β, transforming growth factor beta; TNF, tumor necrosis factor.

### Challenges and future outlook

The most efficient anti-fibrotic treatment approach still remains the elimination of the primary cause of intestinal injury, which would mean nothing less than curing CD. However, since a magic bullet for CD is not and will most likely not be available in the near future, clinical evaluation and optimization of anti-fibrotic drug candidates for stricturing CD is a justifiable and promising treatment strategy.

However, the simple transfer of these agents into CD treatment is premature and multiple obstacles have to be overcome to make use of the above described mediators and mechanisms.

First, the ideal anti-fibrotic drug should target something uniquely expressed in a specific fibrotic complication in a particular organ and should not display any systemic side effects. This is particularly true in case of concomitant injury elsewhere in the body. To date, however, no specific target for intestinal fibrosis or other fibrotic disease has been identified, which supports the hypothesis that mechanisms of fibrosis are shared between different organs.

Second, the optimal timing to commence anti-fibrotic treatment is of utmost importance to CD patients, however, is not defined yet. It is obvious that an early use of anti-fibrotics is expected to be associated with a better outcome in patients prone to this complication. Pre-existing fibrosis and concomitantly increased tissue stiffness can perpetuate fibrosis even in the absence of inflammation. Data from experimental fibrosis seems to confirm that there might be a critical point in evolution of fibrosis when the progress becomes irreversible [184]. Therefore, an early treatment with anti-fibrotics at the same time of anti-inflammatory agents might be mandatory in human CD patients [185]. Unique to CD is a fistulizing disease process and this complication needs to be kept in mind when using drugs inhibiting tissue remodeling because this could theoretically promote fistulizing disease. However, timing may not be simply based on the disease duration, since up to 50% of CD patients present with stricturing or penetrating disease at the time of first diagnosis [186].

Third, a patient-tailored anti-fibrotic treatment approach necessitates the identification of biomarkers to enable individual risk stratifications for stricturing disease and to follow the individual evolution of fibrosis. As opposed to other organs, such as liver [187,188] or lung [159], regrettably, no clinically useful biomarkers for stricturing CD as well as for assessment of therapeutic response are available yet. Promising approaches include serology [189,190] or imaging techniques [191-196], but the currently used tools only detect endpoints based on clinical findings of possible CD fibrosis and hence are not accurate.

Fourth, the best route of application for anti-fibrotic agents in stricturing CD has to be elucidated. In line with the beneficial effects of systemic administration of anti-fibrotics in other organs [24], intravenous or oral application may be appropriate. The CD intestine offers the advantage of potential local therapy as a large proportion of strictures is accessible via endoscopy. Intralesional injection or topical application of anti-fibrotics is a valid and practical therapeutic option, as shown by steroid [197] or infliximab application [198]. This approach may be associated with fewer side effects as compared to systemic administration.

Finally, a general limitation to evaluating potential anti-fibrotic agents is the long-lasting evolution of fibrotic complications in patients with CD requiring clinical trials of long duration and large patient populations, making them very expensive and impractical [24].

It is unclear and in the realm of speculation, which of the reviewed drugs has the highest potential for success as an anti-fibrotic treatment in CD. Combining drugs with a known safety profile and anti-fibrotic efficacy, such as HMG-CoA reductase inhibitors, mTOR inhibitors or inhibitors of the angiotensin system, could serve as a starting point. Given the effect of most substances on a reduction of TGF-β1 proper precaution needs to be taken for carefully monitoring intestinal inflammation. Novel drug delivery systems, such as the Multi Matrix System (MMX®), allowing oral administration of a compound with the substance being released in a defined area of the gastrointestinal tract (such as the terminal ileum) can circumvent systemic side effects, such as hypotension for angiotensin blockade.

**Figure 4:**
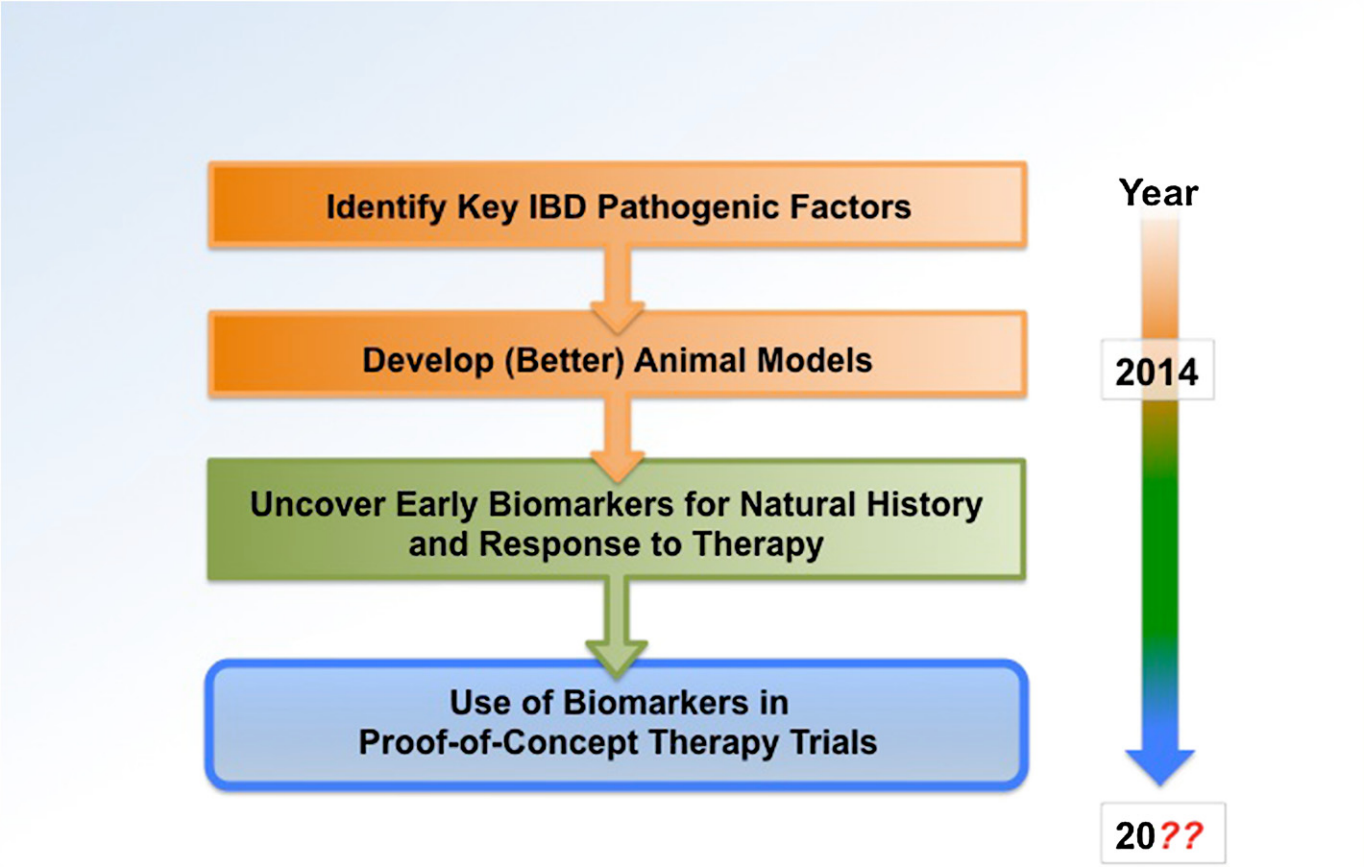
**A suggested path to more efficient translation of new discoveries to Crohn’s disease-associated fibrosis.** Adapted from Friedman *et al*. [24]. IBD, inflammatory bowel disease.

To move forward with specific anti-fibrotic therapies in the future we need to focus on identifying mechanisms of fibrogenesis and to develop new and better animal models for this disease (Figure 4). What, however, is badly missing is the availability of biomarkers for natural history and response to therapy that are readily available and easy to measure. Only through utilization of these markers will the field be able to design clinical studies of reasonable length and sufficient patient number, reflected in affordable budgets that put our ability for testing anti-fibrotic therapies within reach.

## Conclusions

Stricturing CD is still an unresolved problem with strong implications for the patients and a high socioeconomic burden due to frequent hospitalizations and surgery [23]. Despite recent advances in the pathophysiological understanding of fibrosis and significant expansion of the anti-inflammatory armamentarium over the last few decades, the occurrence of intestinal strictures in CD patients did not significantly change. To date, effective therapeutic approaches for stricturing CD are limited to ED or surgical interventions. The data presented in this review highlight the pleiotropic anti-fibrotic actions that have been observed with the use of numerous agents in fibrotic complications of the skin, the lung and the kidney. Thus, it is justified to propose further evaluation of these drug candidates in clinical trials for the management of intestinal fibrosis. However, previous establishment of non-invasive biomarkers to assess the degree of fibrosis, to monitor fibrotic evolution and to predict therapeutic response, combined with the development of imaging techniques to quantify intestinal fibrosis, appear to be essential pre-requisites for individual risk stratification and proper design of clinical trials.

## Abbreviations

6-MP: 6-Mercaptopurine; ACE: Angiotensin-converting enzyme; ALK: Activin receptor-like kinase; AT II: Angiotensin II; AZA: Azathioprine; bFGF: Basic fibroblast growth factor; c-Abl: c-Abelson; CD: Crohn’s disease; CTGF: Connective tissue growth factor; DSS: Dextran sulfate sodium; ECM: Extracellular matrix; ED: Endoscopic dilation; EMT: Epithelial-mesenchymal transition; EndoMT: Endothelial-mesenchymal transition; HMG-CoA: 3-Hydroxy-3-methyl-glutaryl-coenzyme A; IBD: Inflammatory bowel disease; IGF: Insulin-like growth factor; IL: Interleukin; IPF: Idiopathic pulmonary fibrosis; MAPK: Mitogen-activated protein kinase; MMP: Matrix metalloproteinase; mTOR: Mammalian target of rapamycin; NF-κB: Nuclear factor kappa-light-chain-enhancer of activated B cells; NIH: National Institutes of Health; NLM: National Library of Medicine; PDGF: Platelet-derived growth factor; PG-PS: Peptidoglycan-polysaccharide; PPAR: Peroxisome proliferator-activated receptor; RAS: Renin-angiotensin system; ROCK: Rho-associated protein kinase; SAP: Serum amyloid P component; TGF: Transforming growth factor; THBS: Thrombospondin; TIMP: Tissue inhibitor of metalloproteinase; TLR: Toll-like receptor; TNBS: 2,4,6-Trinitrobenzene sulfonic acid; TNF: Tumor necrosis factor; VEGF: Vascular endothelial growth factor.

## Competing interests

The authors declare that they have no competing interests.

## Authors’ contributions

DB and FR developed the review concept, performed the literature review and wrote the manuscript. Both authors read and approved the final version of the manuscript.
